# Developing “SYMBIOTIC” Relationships With Urgent Care Centers: Improving the Referral Process Through Preparedness and Quality Improvement Initiatives

**DOI:** 10.1177/1942602X251403000

**Published:** 2026-01-08

**Authors:** Robert P. Olympia, Neha Gupta, Paige Chardavoyne, Marcus Erdman, Susan J. Boehmer

**Affiliations:** Departments of Emergency Medicine and Pediatrics, Penn State Hershey Medical Center, Hershey, PA, USA; Department of Emergency Medicine, George Washington University, Washington, DC, USA; Department of Psychiatry and Behavioral Medicine, Medical College of Wisconsin, Milwaukee, WI, USA; Department of Pediatrics, US Air Force—673 Medical Group, Joint Base Elmendorf-Richardson, Anchorage, AK, USA; Department of Public Health Services, Penn State Milton S. Hershey Medical Center, Hershey PA, USA

**Keywords:** urgent care center, referral, emergency, school-based health, mental health; school nurse; collaboration

## Abstract

Because of the widespread availability of urgent care centers (UCCs) and the non-emergent nature of many illnesses and injuries occurring in students who attend school, referrals to UCCs may be an option in the management of students without a primary care provider and with certain health insurance providers. Optimizing the care of these students involves several steps for the school nurse: (a) becoming familiar with the capabilities of the UCC in your community, and (b) establishing a collaborative relationship between the school and UCC through closed-loop communication and quality improvement initiatives.

Two students present to your office with the following complaints:

*Case 1: Abigail is a 17-year-old presenting with a 1-cm laceration to her forehead after she tripped during musical rehearsal and hit her head. She did not lose consciousness. She is awake and follows all commands. She complains of a mild generalized headache and dizziness. Her bleeding is well-controlled with pressure.*

*Case 2: Madelyn is a 14-year-old presenting with a sore throat, headache, and abdominal pain. You notice that she has a fever of 104.5°F. She demonstrates no signs of dehydration or altered mental status. She has no difficulty breathing or swallowing and has no neck stiffness. She rates her headache and abdominal pain as 5/10 on a pain severity score.*

Your nursing assessment determines that Abigail and Madelyn would benefit from further evaluation by a healthcare provider. You contact each student’s parent and find out that neither Abigail nor Madelyn has a primary care provider. You are familiar with an urgent care center (UCC) that recently opened in your community. Should the students be evaluated in a UCC or your local emergency department (ED)?

The growth of the urgent care medicine industry over the past few decades can be attributed to the need to seek convenient, high-quality, low-cost care, in response to overcrowding in EDs and limited hours and physician shortages in primary care physician offices (Urgent Care Association, 2023). Where a student can seek care and the cost for that care can be impacted by the type of insurance the student has. For students and families who may have to wait for months for an appointment to establish a primary care provider, a UCC may be a preferable option. Because of the widespread availability of UCCs and the nonemergent nature of many illnesses and injuries occurring in students who attend school, direct referrals to UCCs may be an option in the management of these students (Rothstein, 2021). A questionnaire developed by the authors and distributed to a random group of members of the NASN during the 2021 to 2022 school year determined that 86% of responders reported having a UCC in their community, 79% had directly referred a student to a UCC during the previous school year, and the most common chief complaints of UCC referrals were extremity sprain/strain/contusion, laceration/abrasion, febrile illness, upper respiratory infection, head/neck injury, and vomiting/diarrhea/dehydration.

Optimizing the care of students with nonemergent illness or injury who do not have an established primary care provider involves several steps: (a) becoming familiar with the capabilities of the UCC in your community through preparedness strategies and (b) establishing a collaborative relationship between the school and UCC through closed-loop communication and quality improvement initiatives.

## Becoming Familiar With the UCC’s Capabilities

Services provided by UCCs can be variable (Table 1). School nurses should determine the capabilities of UCCs in their community to appropriately refer students with nonemergent chief complaints. Opportunities to refer students with more emergent chief complaints and mental/behavioral health issues to a UCC have increased over the past few years. Referring students to a UCC who present with more emergent chief complaints (such as abdominal pain, shortness of breath, acute headache, seizure/syncope, and chest pain) may exist as UCCs become more pediatric-focused, employing pediatricians, pediatric advanced practice providers, and emergency medicine trained physicians. Some UCCs have been able to increase their capabilities to perform more sophisticated on-site laboratory/radiologic testing and interventions/procedures traditionally performed in EDs (such as the administration of intravenous fluids, intravenous/nebulized medications, and electrocardiograms; ultrasonography; performing procedures such as procedural sedation and analgesia, reduction/casting/splinting of an extremity fracture, dislocation, or sprains, and complex laceration repair).

**Table 1.** Services Often Offered at Urgent Care Centers

Appropriate chief complaints	Most UCC can manage chief complaints utilizing the “URGENT CARES” mnemonic: **U**-Urinary/vaginal symptoms: Painful urination, increased urinary frequency, blood in urine, vaginal itching/discharge/rash **R**-Rashes (without evidence of altered mental status or shock) **G**-Gastrointestinal complaints: mild abdominal pain, nausea, vomiting, diarrhea, small amount of blood in vomiting and/or stool, mild dehydration **E**-Extremity injuries/mild trauma: sprains, strains, bruises, lacerations, abrasions, head injury without loss of consciousness or altered mental status **N**-Nosebleeds **T**-Temperature: Fever (>100°F) except in children with altered mental status or immunocompromised **C**-Cough and cold symptoms: stuffy or runny nose, sore throat, mild headache **A**-Allergy symptoms: itchy eyes, pink eyes, nasal congestion, sneezing **R**-Respiratory illness: mild asthma attack, pneumonia (fever and cough), excludes those with respiratory distress or significant dehydration **E**-Earaches **S**-Skin infections: cellulitis (red, warm, tender skin), abscesses
Diagnostic testing	On-site laboratory testing may include the following: complete blood counts, inflammatory markers (C-reactive protein, sedimentation rates, procalcitonin), comprehensive metabolic profiles, diabetic testing (hemoglobin A1c), urine pregnancy, urinalysis, rapid strep and throat cultures, rapid viral testing, blood and urine cultures, drug screens, and sexually transmitted infection testing. On-site radiologic testing may include the following: X-rays, ultrasonography, CT scans
Additional services	Additional services may include the following: electrocardiogram; intravenous fluids; oral, intravenous, and nebulized medications; active and passive immunizations; procedural sedation and analgesia; reduction/casting/splinting of an extremity fracture or dislocation or sprains; laceration repair; incision and drainage; foreign body removal; management of epistaxis; adolescent pelvic examinations; on-site radiology and laboratory technologists; telehealth consultation; mental health services; primary care and preventative care visits; sport and employment physical examinations Initial stabilization of adult and pediatric patients who may present with life-threatening medical or traumatic complaints and transfer of these critically ill or injured patients to an ED via Emergency Medical Services

In response to the increasing mental health and addiction crisis affecting children and adolescents (Cushing, 2023), UCCs have begun to employ pediatric psychiatric and behavioral health physicians, therapists, and social workers, and the number of specific mental and behavioral health UCCs have increased throughout the United States to provide screening evaluations, crisis stabilization, safety planning, referrals to providers for treatment, and connections to community resources. Referral to a UCC for a student who is having suicidal or homicidal ideations, acute psychosis, or reported unhealthy or unsafe living situation is not appropriate. Students with these concerns should be seen in an ED.

Given the variety of services that can be offered in a UCC, it is important for the school nurse to be knowledgeable about the type of care that is offered. Helping a student, or a parent/guardian, to select a safe and appropriate setting to seek care is a part of the school nurse’s role in care coordination (National Association of School Nurses, 2024).

## Establishing a Collaborative Relationship Between the School and UCC

Support for students and their families can be enhanced if the school nurse is well acquainted with the available healthcare providers in the community. When there are no primary care providers available or the student does not have an established relationship with a primary care provider, the nurse may suggest that the student be seen for nonemergent illness or injury at a UCC, depending on the type of insurance coverage the student has. Having an established collaborative relationship with the UCC in your community can be accomplished using closed-loop communication and quality improvement initiatives.

While sharing information between the school nurse and the UCC would ensure closed-loop communication, the referral of a student with nonemergent illness or injury would require written consent from the parent or guardian based on Health Insurance Portability and Accountability Act (HIPAA) laws and the Federal Education Rights and Privacy Act (FERPA; Centers for Disease Control and Prevention, 2024). Once written consent is obtained, to allow for bidirectional communication, we suggest that school staff and UCC staff develop a system to ensure that the most important information regarding the student and their illness/injury is communicated. The authors have created mnemonics to assist school and UCC staff relay this important information—the “SYMBIOTIC” mnemonic to relay information from the school to the UCC (Table 2) and the “DISPO” mnemonic to relay information back to the school from the UCC (Table 3). We suggest that schools and UCCs utilize electronic or written forms throughout the school referral process and UCC disposition process, incorporating these two mnemonics.

**Table 2.** “SYMBIOTIC” Mnemonic With Applications to Case 1 and Case 2 (Developed by Authors, Information to be Relayed From School Nurse to Urgent Care Center)

Mnemonic	Meaning	Case 1	Case 2
S	Signs and symptoms—what is the student presenting with?	Headache, dizziness, forehead laceration	Fever, sore throat, headache, abdominal pain
Y	“Why” are you referring the student to the urgent care center? What is your concern?	Concern for a concussion, intracranial bleeding, needing laceration repair	Concern for strep throat, maybe meningitis or appendicitis
M	Medications and allergies	No medications or allergies	Epinephrine autoinjector as needed
B	Biography (Student’s past medical and past surgical history)	History of a previous concussion 1 year ago	History of asthma and food allergies
I	Interventions (Have you given any medications or performed any procedures prior to referral?)	Oral acetaminophen per standing order, pressure dressing applied to laceration	Oral acetaminophen per standing order
O	Observers (Any staff or students observe the mechanism of injury or event surrounding the presenting complaint?)	Observed by other musical cast members and her director	Not applicable
T	Timeline (Sequential events prior to the referral)	Brought to the nurse’s office shortly after the fall	Brought to the nurse’s office by her teacher after complaining of severe headache and abdominal pain
I	Injuries (specific mechanism of injury and injuries sustained, if applicable)	Fall from a standing position onto the floor striking her head	Not applicable
C	Contact information (School nurse name, phone number, email address, etc.) to provide follow-up	[Your information here]	[Your information here]

**Table 3.** “DISPO” Mnemonic With Applications to Case 1 and Case 2 (Developed by Authors, Information to be Relayed From Urgent Care Center to School Nurse)

Mnemonic	Meaning	Case 1	Case 2
D	Differential diagnosis—based on the initial history, presenting signs and symptoms, and physical examination, what was UCC provider concerned about?	Concussion vs. intracranial injury, laceration requiring repair	Strep pharyngitis, viral infection, less likely appendicitis
I	Interventions—were there any diagnostic testing (laboratory or radiologic studies), medications given, or procedures performed?	No CT scan of the brain required, routine laceration repair with sutures	Rapid strep test (positive), complete blood count (within normal limits), urinalysis (within normal limits)
S	Suspicion—based on interventions performed, what is the most likely diagnosis	Concussion, forehead laceration	Strep pharyngitis causing headache and abdominal pain (mesenteric adenitis)
P	Plan—is there care that the student needs based on the suspicion, such as medications, follow-up care.	Postconcussion care, wound care, acetaminophen or Ibuprofen for pain, suture removal in 5 days	Started on a 10-day course of amoxicillin, acetaminophen or ibuprofen for pain
O	Outcome—what is the student’s disposition (immediate transfer to an ED, discharge home, etc.)	Discharge home with Parent	Discharge home with Parent

Data based on UCC referrals should be collected and analyzed. Schools and UCCs should institute quality improvement initiatives regularly to measure the appropriateness of referrals to UCCs, to determine the outcomes of students with emergent and nonemergent illness or injury who are referred to a UCC, and to identify barriers in the referral process. Identified knowledge gaps should lead to educational opportunities. For example, if through data analysis a school determines that a significant number of students referred to UCCs with cough and cold symptoms are subsequently referred to the ED with respiratory distress, quality improvement initiatives may result in continuing educational opportunities for school nurses to learn about signs and symptoms of pneumonia and signs of respiratory distress.

UCCs are increasing in numbers throughout the United States, and their ability to provide convenient, high-quality, low-cost care, in response to overcrowding in EDs and limited hours and physician shortages in primary care physician offices make their services valuable within a health care system. Current relationships between EDs, UCCs, and school nurses/staff should be assessed, UCC capabilities discussed, and deficiencies improved through education and quality improvement initiatives. In addition, strategies to strengthen closed-loop communication between UCCs and school nurses/staff should be emphasized (utilizing methods of communication, such as the “SYMBIOTIC” and “DISPO” mnemonics presented here), focusing on the appropriateness of referrals and the coordination of follow-up for students who are referred. Future studies should focus on the appropriateness of UCC referrals from schools, the outcome of students referred to the UCC, the effect that UCC referrals from schools have on ED overcrowding, and the utilization of UCCs by schools based on their region, size, or student density.

## References

[bibr1-1942602X251403000] Centers for Disease Control and Prevention. (2024, July 10). Health information & privacy: FERPA and HIPAA. Public Health Law Program. https://www.cdc.gov/phlp/php/resources/?CDC_AAref_Val=https://www.cdc.gov/phlp/publications/topic/healthinformationprivacy.html

[bibr2-1942602X251403000] Cushing A. M. Liberman D. B. Pham P. K. Michelson K. A. Festekjian A. Chang T. P. Chaudhari P. P. (2023). Mental health revisits at US pediatric emergency departments. JAMA Pediatrics, 177(2), 168–176. 36574251 10.1001/jamapediatrics.2022.4885 PMC9856860

[bibr3-1942602X251403000] National Association of School Nurses. (2024). A contemporary framework update for today’s school nursing landscape: Introducing the *School Nursing Practice Framework*. NASN School Nurse, 39(3), 140–147. https://www.doi.org/10.1177/1942602X241241092 38623932 10.1177/1942602X241241092

[bibr4-1942602X251403000] Rothstein R. Zhen K. Kim R. Y. Olympia R. P. (2021). Acuity-appropriate triage of chief complaints found on urgent care center organization websites. American Journal of Emergency Medicine, 43, 276–280. 33010993 10.1016/j.ajem.2020.06.050

[bibr5-1942602X251403000] Urgent Care Association. (2023). Urgent care industry white paper: The essential nature of the urgent care in the healthcare ecosystem post-COVID-19. https://urgentcareassociation.org/wp-content/uploads/2023-Urgent-Care-Industry-White-Paper.pdf

